# Health related quality of life among Rheumatic Fever and Rheumatic Heart Disease patients in India

**DOI:** 10.1371/journal.pone.0259340

**Published:** 2021-10-29

**Authors:** Jyoti Dixit, Gaurav Jyani, Shankar Prinja, Yashpaul Sharma

**Affiliations:** 1 Department of Community Medicine and School of Public Health, Post Graduate Institute of Medical Education and Research, Chandigarh, India; 2 Department of Cardiology, Advanced Cardiac Centre, Postgraduate Institute of Medical Education and Research, Chandigarh, India; University of Western Australia, AUSTRALIA

## Abstract

**Background:**

Measurement of health-related quality of life (HRQOL) of people with chronic illnesses has become extremely important as the mortality rates associated with such illnesses have decreased and survival rates have increased. Thereby, such measurements not only provide insights into physical, mental and social dimensions of patient’s health, but also allow monitoring of the results of interventions, complementing the traditional methods based on morbidity and mortality.

**Objective:**

The present study was conducted to describe the HRQOL of patients suffering from Rheumatic Fever (RF) and Rheumatic Heart Disease (RHD), and to identify socio-demographic and clinical factors as predictors of HRQOL.

**Methodology:**

A cross-sectional study was conducted to assess the HRQOL among 702 RF and RHD patients using EuroQol 5-dimensions 5-levels instrument (EQ-5D-5L), EuroQol Visual Analogue Scale and Time Trade off method. Mean EQ-5D-5L quality of life scores were calculated using EQ5D index value calculator across different stages of RF and RHD. Proportions of patients reporting problems in different attributes of EQ-5D-5L were calculated. The impact of socio-economic determinants on HRQOL was assessed.

**Results:**

The mean EQ-5D-5L utility scores among RF, RHD and RHD with Congestive heart failure patients (CHF) were estimated as 0.952 [95% Confidence Interval (CI): 0.929–0.975], 0.820 [95% CI: 0.799–0.842] and 0.800 [95% CI: 0.772–0.829] respectively. The most frequently reported problem among RF/RHD patients was pain/discomfort (33.8%) followed by difficulty in performing usual activities (23.9%) patients, mobility (22.7%) and anxiety/depression (22%). Patients with an annual income of less than 50,000 Indian National Rupees (INR) reported the highest EQ-5D-5L score of 0.872, followed by those in the income group of more than INR 200,000 (0.835), INR 50,000–100,000 (0.832) and INR 100,000–200,000 (0.828). Better HRQOL was reported by RHD patients (including RHD with CHF) who underwent balloon valvotomy (0.806) as compared to valve replacement surgery (0.645).

**Conclusion:**

RF and RHD significantly impact the HRQOL of patients. Interventions aiming to improve HRQOL of RF/RHD patients should focus upon ameliorating pain and implementation of secondary prevention strategies for reducing the progression from ARF to RHD and prevention of RHD-related complications.

## Background

Health related quality of life (HRQOL) encompasses the physical, psychological, and social domains of health, seen as distinct areas that are influenced by a person’s experiences, beliefs, expectations, and perceptions [1]. Over the past few decades, there has been an increasing predominance of chronic disorders resulting from improvement in living conditions and medical technology, proper hygiene, better prevention and management of infectious diseases and overall aging of the population. Currently, there are high numbers of people living with chronic diseases which can adversely affect their quality of life [2–4]. The majority of chronic diseases hold the potential to worsen the overall health of patients by limiting their capacity to live well, limit productivity as well as HRQOL and thus are major contributors to health care costs [5].

Rheumatic Heart Disease (RHD), a sequela to Rheumatic Fever (RF), is one of the common chronic disorders affecting nearly 20 million people in developing countries [6]. The disease is strongly associated with poverty, poor living conditions and limited access to health care. Global disease estimates in 2005 reported 471,000 RF cases annually across the world, which largely occurred in children aged 5–15 years [6]. India is home to 40% of all people living with RHD. Of the estimated 33 million people with RHD, 13.2 million live in India. Likewise, in 2015, of the 347 000 deaths due to RHD worldwide, over a third are estimated to have occurred in India [7]. The prognosis of patients with RHD is very poor in rural India [8, 9]. The beginning of this chronic state usually results in devastating symptoms and physical presentations, all contributing to poor quality of life in these patients. The literature also suggests that RHD exerts a negative impact on HRQOL [10–13].

Despite the increased survival associated with advances in medical technology, problems such as organ dysfunction [14, 15], psychosocial disorders, and effects on neurological development might still occur, limiting patients’ cognitive development and their productivity [10, 16, 17]. The presence of cardiomyopathies and long-term prophylactic treatment with injections of benzathine penicillin often results in psychological disorders and suboptimal treatment adherence, thereby negatively influencing the HRQOL. [18]. Therefore, HRQOL is an important measure to evaluate the impact of a disease and the effects of medical interventions and drug therapies on people with chronic illnesses. In this way, measurement of HRQOL provides an opportunity for health services to be modified to become more patient-centric [19].

Usually, two types of instruments, namely *generic* and *specific*, can be used to measure HRQOL [20]. The generic instruments are used to collect information on healthy and ill individuals at the population level and allow for the comparison of HRQOL across different conditions and between healthy and ill individuals [21, 22]. In contrast, disease-specific instruments aim to collect information on health problems that are more specific to a particular disease [21, 22]. The generic instruments used to measure HRQOL among RF and RHD patients include Pediatric Quality of Life Inventory™ (PedsQL™ 3.0) Cardiac Module, Child Health Questionnaire Parent Form—CHQ-PF50 and CHQ-PF28 [23, 24]. The most commonly used generic instruments for measuring the HRQOL are the EuroQol-5 Dimensional 5 Levels instrument (EQ-5D-5L), Short Form-6 Dimension (SF-6D), and Health Utilities Index Mark 2 and Mark 3 (HUI2/3) [25–27]. The generic preference-based measures of HRQOL are commonly used in the Health Technology Assessment (HTA) studies, as they provide a multidimensional description of health that is combined with survival to generate quality-adjusted life-years (QALYs). The QALY is an outcome used in the cost utility analysis method of economic evaluation [28, 29].

Against this background, the present study aimed to assess HRQOL of RF and RHD patients who were subjected to a range of treatment approaches, including secondary prophylaxis, conservative management and surgical intervention. We aimed to compare the HRQOL using a range of methods, including the EQ-5D-5L, the EuroQoL Visual Analogue Scale (EQ-VAS) and time trade off (TTO) [30, 31].

## Methodology

**Fig 1:**
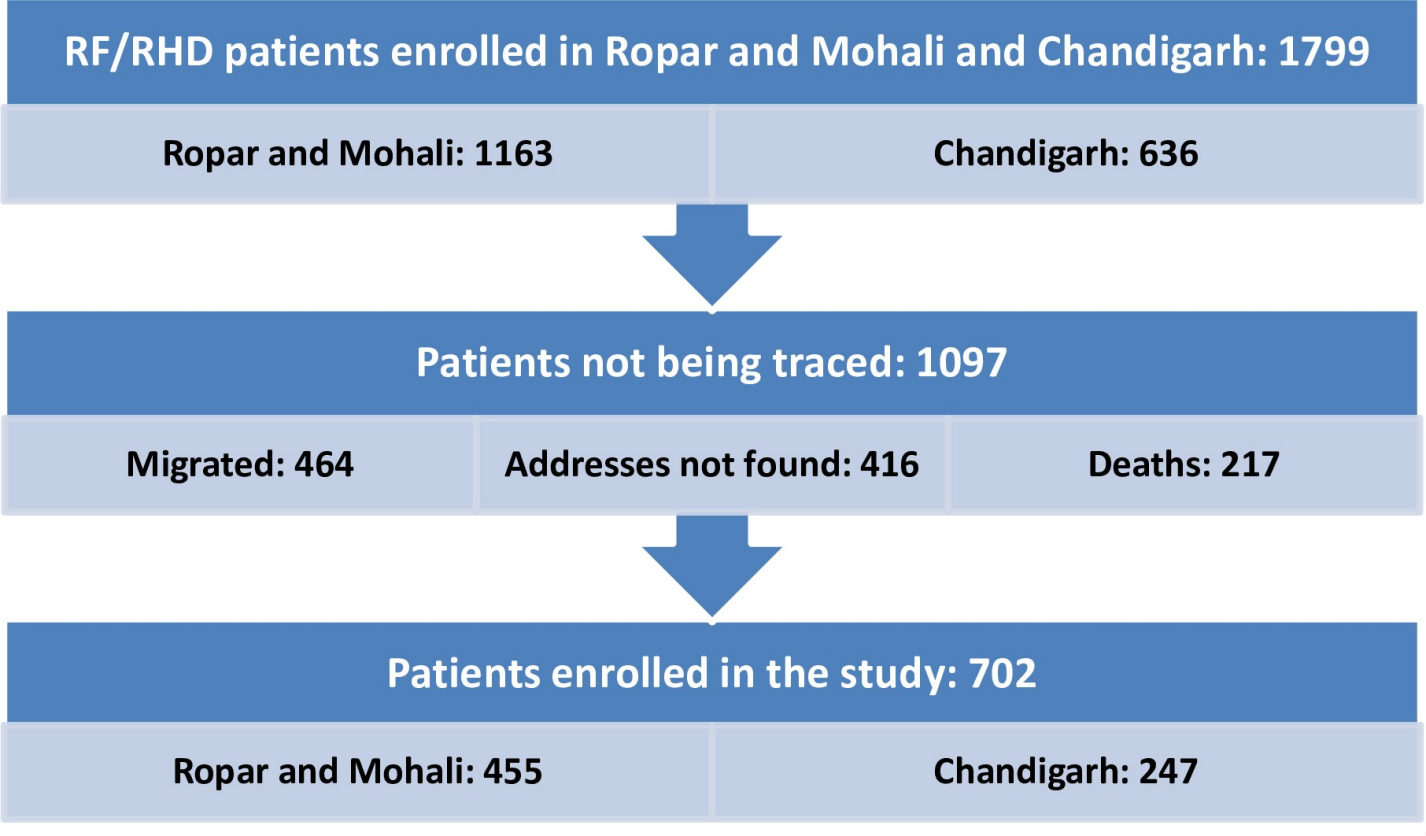
Flowchart showing process of sample selection.

A cross-sectional study was conducted to assess the HRQOL of 702 patients suffering from RF and RHD, enrolled in a population-based RF/RHD registry covering two districts of Punjab (Ropar and Mohali) and one union territory, Chandigarh. The patients were interviewed using the EQ-5D-5L, EQ-VAS and TTO tools and followed up for a period of one year. Detailed sample selection is shown in Fig 1.

### Description of items in EQ-5D-5L tool, EQ-VAS and TTO for HRQOL assessment

EQ-5D-5L is a generic questionnaire consisting of five attributes: mobility, self-care, usual activity, pain/discomfort and anxiety/depression [32]. Each of these attributes has five levels: no problems, slight problems, moderate problems, severe problems and extreme problem. The EQ-5D health state is converted into a utility score using value set which is a country-specific scoring algorithm. EQ-5D was used to compute a single utility score ranging between <0 and 1 on the basis of individuals’ responses to questions regarding the impact of RF and RHD on their lives. The possible health states that could be defined was 3125 (5^5^), along with ‘unconscious’ and ‘dead’ state making a total of 3127 states [33]. A utility score of ‘1’ implies perfect health and ‘0’ implies death with a range of 1 to -0.549 [34]. The negative value of utility score represents health state worse than death. We used the reference population value set from Thailand to compute the HRQOL index value of individual health states. Given the absence of Indian tariff values, this is in accordance with recommendations made in the draft Indian reference case, developed by ‘Health Technology Assessment in India’ (HTAIn) for conducting HTA in India [35–38].

In addition, all the patients were asked to rate their present health state between 0–100 using EQ-VAS [25]. The word “visual” in the term visual analogue scale (VAS) emphasizes the concrete nature of this type of scale (straight line), in contrast to abstract, non-representable evaluation scales (for example-I am unable to walk). A VAS is usually a 100-mm long horizontal line ranging from 0 to 100 at each end to express the extremes of the feeling wherein 0 represents the worst imaginable health state and 100 the best. The patients mark the point on the line that best corresponds to their present health state. They are instructed to put a cross on the straight line at the point that most accurately expresses their degree of agreement.The scores represent the ordinal rankings of the health outcomes, where ‘0’ denotes the worst health state and ‘100’ denotes the best health state from the patients’ perspective [25].

The third method used for HRQOL assessment was TTO wherein the preferences of each respondent for a specific health state are elicited by asking the respondent to choose between two different health states, each assigned a specific number of years followed by death. In the present study, participants were asked to choose between either living a longer life (a remaining life expectancy of 10 years) with same diseased state or living a shorter life (< 10 years) without having the current health condition. There are various methodological differences between these measures as utility scores derived from direct (TTO and VAS) and indirect measures (EQ-5D-5L) represent different respondent perspectives. The direct methods like TTO are used to capture values that patients assign to their own health state while VAS is simply based on rating exercise. However, indirect measures like EQ-5D-5L use published tariffs to assign values of the general population to patients’ description of their health. EQ-VAS and TTO provide information that is complementary to the EQ-5D profile. For instance-patients who report no problems in EQ-5D tool tend to rate their health less than 100 on VAS. Similarly, TTO helps in capturing the patient’s perspective about their own health state. Thus, the present study has used all three measures for HRQOL assessment of RF/RHD patients.

#### Data collection

The EQ-5D-5L tool was administered to all 702 RF and RHD patients by trained field investigators. The present study has elicited the HRQOL directly from the patients and no proxies were used. House-to-house visits were made in order to conduct face-to-face interviews, which lasted for average 20–25 minutes. The information related to socio-demographic characteristics, disease condition whether RF/RHD/RHD with CHF, type of intervention such as secondary prophylaxis/conservative management/both conservative management and secondary prophylaxis/surgical intervention (balloon valvotomy or valve replacement surgery) were collected, allowing categorisation into 9 disease and intervention groups.

### Disease and intervention categories

1. ***Rheumatic Fever Remission on ‘No intervention’(N = 80)*:** This refers to patients diagnosed with rheumatic fever as per modified Jones criteria [39], among whom treatment (secondary prophylaxis) was stopped by the doctor due to cessation of RF symptoms and completion of secondary prophylaxis regime according to guidelines. These patients were referred as rheumatic fever remission cases as RF symptoms were subsided among them.
2. ***Rheumatic Fever on ‘Secondary Prophylaxis (N = 19)*:** This refers to patients suffering from rheumatic fever diagnosed on the basis of modified Jones criteria [39] and who were active treatment i.e. on secondary prevention i.e. 4-weekly Inj. Benzathine penicillin.
3. ***Rheumatic Heart Disease on ‘Secondary Prophylaxis’ (N = 45)*:** This refers to the patients who had rheumatic carditis and were on secondary prophylaxis i.e. 4-weekly Inj. Benzathine penicillin.
4. ***Rheumatic Heart Disease on ‘Conservative treatment’ (N = 153)*:** This refers to the patients suffering from RHD diagnosed using echocardiography and on medical management.
5. ***Rheumatic Heart Disease on ‘Secondary prophylaxis and Conservative management (N = 22)*:** This refers to the patients suffering from RHD diagnosed using echocardiography and who were on two interventions namely secondary prophylaxis and medical management.
6. ***Severe Rheumatic Heart Disease (N = 22)*:** This refers to the patients suffering from RHD having valvular lesions who were being advised surgery by the doctor but they did not avail it and were on medical management.
7. ***Rheumatic heart disease with Congestive heart failure on ‘Conservative treatment’ (N = 148)*:** This refers to the patients suffering from RHD along with congestive heart failure as a complication and who were on medical management.
8. ***Rheumatic heart disease on ‘valve replacement surgery’ (N = 32)*:** This refers to the RHD patients who had valvular lesions, were advised surgery and underwent valve replacement surgery.
9. ***Rheumatic heart disease on ‘balloon valvotomy’ (N = 68)*:** This refers to the RHD patients who had valvular lesions, were advised and underwent balloon valvotomy.

#### Data analysis

The EQ-5D-5L, EQ-VAS and TTO data were analysed using Microsoft Excel and Statistical Package for Social Sciences (SPSS) for windows version 21.0 Chicago, SPSS Inc. Percentages of patients reporting problems in each attribute of EQ-5D-5L were calculated. Mean stage-specific utility scores for patients falling into different categories namely RF, RHD, RHD with congestive heart failure (CHF) were calculated. In order to assess the impact of intervention, intervention-specific utility scores were also computed for each disease stage using EQ-5D-5L, EQ-VAS and Time Trade Off (TTO) methods. The description of the nine disease and intervention categories is given below:

### Difference of HRQOL among patients of different socio-demographic characteristics

The difference in the mean HRQOL scores among the patients of different socio- demographic characteristics was assessed using the statistical tools ANOVA, independent samples t- test and multiple linear regression. ANOVA was used to assess the difference of mean HRQOL scores among patients of different age, religion, residence, education, annual household income and clinical severity, whereas independent samples t-test was used to investigate the difference across gender. Multiple linear regression was used to assess the determinants of HRQOL. The regression equation so formed can be written as:

$$y=\alpha +\beta_{1}\alpha_{1}+\beta_{2}\alpha_{2}+\beta_{3}\alpha_{3}+\beta_{4}\alpha_{4}+\beta_{5}\alpha_{5}+\beta_{6}\alpha_{6+}\beta_{7}\alpha_{7+}\beta_{8}\alpha_{8+}\beta_{9}\alpha_{9}$$

Where α is constant, α_1_–α_9_ denote gender, religion, annual household income, clinical severity, residence, education, occupation, marital status and age (independent variables), β_1_–β_6_ are regression coefficients for all the independent variables, and y is mean EQ-5D-5L score (independent variable).

#### Ethical considerations

The study complies with the Declaration of Helsinki and an ethical approval to undertake the study was obtained from Institute Ethics Committee of the Postgraduate Institute of Medical Education and Research (PGIMER), Chandigarh. Informed written consent was obtained from all the study participants. All participants above the age of 18 years gave the consent for themselves, while parental or guardian consent was sought for the participants below the 18 years of age.

## Results

The HRQOL of 702 patients was assessed using EQ-5D-5L, EQ-VAS and TTO methods. The majority of patients were in the 15–30 years age group (41%) followed by 30–45 years (31.6%) and 45–60 years (20.8%). The disease was found to be more prevalent among females (58.1%). More than half of the participants were residing in rural areas (51%) followed by urban areas (47.7%) with only 1.3% in slums. Just over two-thirds were married and just under one-third were unmarried, with 2% being widowed/separated from their spouses. Almost t\wo thirds (64%). The 36% unemployed people included regular salaried/wage employees (18%), wage labourers (7%), own account workers (6%) and cultivators (3%). The majority of the patients were found to be suffering from RHD (54.3%), followed by RHD with CHF (31.6%) and RF (14%). Detailed sample characteristics are summarised in Table 1.

**Table 1:** EQ-5D-5L scores among different socio-demographic groups of Rheumatic fever and Rheumatic heart disease patients.

Characteristics	Number of patients (Percentage)	Mean EQ5D score (95% CI)	p value
**Age in years**			
Up-to 15 years	14 (1.99)	0.920 (0.840–0.999)	0.035
15–30	288 (41.0)	0.856 (0.833–0.879)	
30–45	222 (31.6)	0.814 (0.787–0.840)	
45–60	146 (20.8)	0.806 (0.764–0.847)	
More than 60	32 (4.61)	0.834 (0.768–0.900)	
**Gender**			
Male	294 (41.9)	0.854 (0.831–0.878)	0.018
Female	408 (58.1)	0.817 (0.796–0.837)	
**Religion**			
Hindu	420 (59.8)	0.829 (0.809–0.849)	0.814
Muslim	14 (1.99)	0.818 (0.693–0.944)	
Sikh	268 (38.21)	0.839 (0.814–0.863)	
**Residential Status**			
Urban	336 (47.7)	0.833 (0.811–0.856)	0.767
Rural	357 (51.0)	0.833 (0.811–0.855)	
Slum	9 (1.3)	0.782 (0.596–0.967)	
**Educational status**			
Illiterate	103 (14.7)	0.785 (0.742–0.827)	0.017
Primary and Middle	168 (23.9)	0.830 (0.799–0.862)	
Matric and Senior Secondary	312 (44.5)	0.856 (0.834–0.878)	
Graduate and Postgraduate	119 (16.9)	0.815 (0.773–0.856)	
**Occupation**			
Employed	250 (35.6)	0.837 (0.810–0.864)	0.646
Unemployed	452 (64.4)	0.830 (0.811–0.849)	
**Marital status**			
Unmarried	213 (30.3)	0.863 (0.838–0.889)	
Married	476 (67.8)	0.820 (0.801–0.840)	0.026
Widow/ Separated/ Divorced	13 (1.9)	0.775 (0.659–0.890)	
**Annual household income (INR)**			
Less than 50,000	12 (1.7)	0.872 (0.766–0.979)	0.897
50,000–1 lac	181 (25.9)	0.832 (0.802–0.861)	
1 lac- 2 lac	249 (35.4)	0.828 (0.801–0.855)	
More than 2 lac	260 (37)	0.835 (0.809–0.861)	
**Clinical severity**			
Rheumatic Fever	99 (14.1)	0.952 (0.929–0.975)	0.000
Rheumatic Heart Disease	381 (54.3)	0.820 (0.799–0.842)	
Rheumatic Heart Disease with Congestive heart failure (CHF)	222 (31.6)	0.800 (0.772–0.829)	

### Socioeconomic variations of the EQ-5D-5L index among RF/RHD patients

The mean EQ-5D-5L indices by socioeconomic groups of RF and RHD patients are presented in Table 1. Males were found to have a higher HRQOL (EQ-5D-5L) index (0.854 [95% CI: 0.831–0.878]) compared to females (0.817 [95% CI: 0.796–0.837]). The highest mean EQ-5D-5L score among patients aged 15 years old or less was 0.920 [95%CI: 0.840–0.999]. All scores were lower in the older age groups, including 0.856 [95%CI:0.833–0.879], 0.814 [95%CI: 0.787–0.840], 0.806 [95%CI: 0.764–0.847] and 0.834 [95% CI: 0.768–0.900] among patients aged 15–30 years, 30–45 years, 45–60 years and 60 years and above respectively.

Higher HRQOL was observed among RF/RHD patients of urban and rural areas (0.833 and 0.833 respectively) as compared to those of slum area (0.782). Furthermore, HRQOL of RF and RHD patients were found to be highest (0.872) among lowest income group having an annual household income of less than INR 50,000 Indian National Rupees (USD 67 or less). This is followed by those in the income group of more than INR 200,000 (USD 2683 and above), INR 50,000–100,000 (USD 67 to 1341) and INR 100,000–200,000 (USD 1341 to 2683) reporting EQ-5D-5L scores of 0.835, 0.832 and 0.828 respectively. (Table 1)

The mean EQ-5D-5L utility scores among RF, RHD and RHD with CHF patients were estimated as 0.952 [95% CI: 0.929–0.975], 0.820 [95% CI: 0.799–0.842] and 0.800 [95% CI: 0.772–0.829] respectively. The HRQOL decreased with the increase in clinical severity (p value < 0.001). However, there were no differences observed among the mean EQ-5D-5L scores within these groups (age, gender, marital status, religion, residence, education, occupation and annual household income). (Table 1)

Results of multiple linear regression implied that even after controlling the socio-demographic variables, HRQOL of patients decreased with the increase in the clinical severity of the disease. However, the other independent variables considered in the regression model were not found to be the significant predictors for HRQOL. (Table 2)

**Table 2:** Determinants of health related quality of life (EQ-5D-5L score) among RF/RHD patients.

Variable	Beta	95% confidence interval	p value
Age	-0.017	-0.026 to 0.018	0.730
Gender	-0.049	-0.060 to 0.019	0.310
Marital status	-0.017	-.0.049 to 0.035	0.744
Religion	-0.002	-0.031 to 0.030	0.953
Locality	-0.052	-0.054 to 0.012	0.210
Education	0.001	-0.019 to 0.019	0.978
Occupation	-0.013	-0.045 to 0.034	0.783
Annual income	-0.004	-0.022 to 0.019	0.918
Clinical severity	-0.182	-0.085 to -0.032	**0.000**

### Quality of life assessment using EQ-VAS and TTO

Mean EQ-VAS utility scores for RF, RHD and RHD with CHF were estimated as 0.940 [95% CI: 0.927–0.953], 0.840 [95% CI: 0.828–0.853] and 0.798 [95% CI: 0.785–0.811] respectively. Mean TTO utility score for RF, RHD and RHD with CHF were estimated as 0.897 [95% CI: 0.883–0.911], 0.837 [95% CI: 0.829–0.843] and 0.823 [95% CI: 0.815–0.830] respectively.

### Distribution of RF and RHD patients by EQ-5D-5L

Table 3 describes the distribution of RF and RHD patients according to EQ-5D-5L dimensions and levels. Across the five dimensions, pain/discomfort is the most reported problem and was 1.5 to 3 times more frequently reported (33.8%) than the other dimensions, followed by difficulty in performing usual activities reported by 23.9% patients. While 22.7% RF/RHD patients reported to have problems in walking about, 22% patients had anxiety/depression. One in ten (10.1%) patients reported to have difficulties in activities pertaining to self-care.

**Table 3:** Self-reported problems by patients suffering from Rheumatic Fever and Rheumatic Heart Disease in five domains of EQ-5D-5L.

Domains of EQ-5D-5L	Mobility	Self-Care	Usual Activities	Pain/Discomfort	Anxiety/Depression
	N (%)	N (%)			
			N (%)	N (%)	N (%)
**No Problem**	542 (77.2%)	631 (89.9%)	534 (76.1%)	465 (66.2%)	548 (78.1%)
**Slight problem**	116 (16.5%)	57 (8.1%)	120 (17.1%)	183 (26.1%)	125 (17.8%)
**Moderate Problem**	34 (4.8%)	10 (1.4%)	31 (4.4%)	45 (6.4%)	21 (3%)
**Severe Problem**	7 (1%)	4 (0.6%)	15 (2.1%)	9 (1.3%)	4 (0.6%)
**Extreme Problem**	3(0.4%)	0 (0%)	2 (0.3%)	0 (0%)	4 (0.6%)

Stage specific mean EQ-5D-5L, EQ-VAS and TTO scores based on various interventions along with confidence intervals are presented in Table 4. Better HRQOL was reported by RF patients on ‘No intervention’ (referred as RF remission) using EQ-5D-5L dimensions as compared to RF patients on secondary prophylaxis (0.955 versus 0.937). The HRQOL scores of RHD patients on secondary prophylaxis, conservative treatment and both secondary prophylaxis and conservative treatment using EQ-5D-5L dimensions were estimated as 0.888, 0.795 and 0.742 respectively. The HRQOL among RHD patients was found to be decreasing with increase in intensity of the intervention as per clinical severity of the disease. Better HRQOL was reported by RHD patients (including RHD with CHF) who underwent balloon valvotomy (0.806) as compared to valve replacement surgery (0.645).

**Table 4:** Stage-wise quality of life scores of patients suffering from Rheumatic Fever and Rheumatic Heart Disease on the basis of different interventions.

Disease	No. of patients	Intervention	Health related quality of life score		
			Mean EQ-5D-5L Score* (95% C.I.)	Visual analogue scale (VAS) score (95% C.I.)	Time Trade off (TTO) Score (95% C.I.)
Rheumatic Fever Remission	80	No intervention**	0.955 (0.927–0.979)	0.945 (0.930–0.959)	0.915 (0.903–0.928)
Rheumatic Fever	19	Secondary prophylaxis	0.937 (0.886–0.977)	0.921 (0.888–0.952)	0.821 (0.774–0.858)
Rheumatic heart disease	45	Secondary prophylaxis	0.888 (0.846–0.930)	0.860 (0.828–0.888)	0.824 (0.802–0.847)
	153	Conservative Rx.	0.795 (0.763–0.826)	0.801 (0.784–0.819)	0.850 (0.840–0.860)
	22	Secondary prophylaxis and Conservative Rx.	0.742 (0.648–0.839)	0.799 (0.745–0.851)	0.832 (0.804–0.859)
Rheumatic Heart Disease (Severe)	22	Conservative treatment.; Surgery advised but not availed	0.658 (0.562–0.758)	0.743 (0.713–0.775)	0.820 (0.768–0.859)
Rheumatic Heart Disease with Congestive Heart Failure	148	Conservative treatment	0.7860 (0.755–0.817)	0.797 (0.779–0.814)	0.834 (0.824–0.845)
Rheumatic Heart Disease	32	Valve Replacement Surgery	0.645 (0.584–0.712)	0.779 (0.741–0.813)	0.839 (0.815–0.860)
	68	Balloon Valvotomy	0.806 (0.756–0.859)	0.879 (0.857–0.900)	0.854 (0.837–0.871)

* EuroQol 5 Dimensional score, values derived from Thailand tariff values.

**Treatment stopped by doctor.

## Discussion

The present study is the first in India to measure HRQOL of RF and RHD patients using EQ-5D-5L, VAS and TTO methods. The majority of participants were in the 15–30 years age group (41%) followed by 30–45 years (31.6%) and 45–60 years (20.8%). Thus, it clearly states that RF and RHD continues to affect patients throughout the life and affects most productive years of life. The mean EQ-5D utility score among RF, RHD and RHD with CHF patients was estimated as 0.952 [95% CI: 0.929–0.975], 0.820 [95% CI: 0.799–0.842] and 0.800 [95% CI: 0.772–0.829] respectively. Mean EQ-VAS utility scores for RF, RHD and RHD with CHF were estimated as 0.940 [95% CI: 0.927–0.953], 0.840 [95% CI: 0.828–0.853] and 0.798 [95% CI: 0.785–0.811] respectively. Mean TTO utility score for RF, RHD and RHD with CHF were estimated as 0.897 [95% CI: 0.883–0.911], 0.837 [95% CI: 0.829–0.843] and 0.823 [95% CI: 0.815–0.830] respectively. We also found a declining trend in EQ-5D-5L, EQ-VAS and TTO utility scores across RF, RHD and RHD with CHF disease states. These findings are in line with other studies, as well as biological understanding of the disease, that HRQOL of patients declines as the disease progresses [40, 41]. The study findings also revealed that utility scores using TTO are lower as compared to VAS in less severe stages (RF, RF remission and RHD on secondary prophylaxis). However, as the clinical severity increases, the opposite trend is found, resulting in higher TTO utility scores as compared to VAS. This implies that patients are willing to trade off fewer years in severe stages although report lower quality of life on VAS [42–44]. In the context of surgical intervention, better HRQOL has been reported among patients who underwent balloon valvotomy (0.806) as compared to valve replacement surgery (0.645) as balloon valvotomy is advised in less severe stages of RHD contrary to valve replacement wherein condition is more critical.

Measurement of utility scores for RF and RHD has been attempted in various countries among children aged 5–18 years using other HRQOL assessment tools such as SF-36, CHQ, PedsQL™ 3.0 Cardiac Module [45, 46]. However, most studies used parents as proxy for valuation of quality of life among children, despite parents of children with heart diseases known to report significantly poorer HRQOL than the children themselves [47]. None of these have assessed the HRQOL of RF, RHD and RHD associated with CHF using EQ-5D-5L tool and thus cannot be compared with our findings. The most commonly reported problem by RF and RHD patients in India was found to be pain/ discomfort which is also consistent with findings of other studies [40, 41]. This implies that interventions aiming to improve HRQOL of RF and RHD patients should be more patient-focussed and should give more attention to this aspect in order to achieve better patient outcomes. Further, the present study found that the HRQOL of RF/RHD patients is primarily determined by the clinical severity as study findings showed the declining trend in HRQOL with increase in severity of the disease. Thus, the interventions focussing on reducing the progression of RF to RHD and the prevention of RHD-related complications would have a significant impact on quality of life. In order to achieve this, early diagnosis and secondary prophylaxis is the key intervention for treatment of RF patients as these reduce the progression of ARF to RHD [48].

In this regard, various initiatives had been taken by the Government of India, such as launch of *Rashtriya Bal Swasthaya Karyakram* (RBSK) in 2013 [49]. Screening and early intervention services under RBSK scheme aim to identify 30 health conditions, including RHD, to provide free treatment for children from birth to 18 years of age. Another such initiative is the establishment of RF/RHD registries at primary and secondary level of health-care facilities [50]. These registries aim to register RF/RHD patients to provide them with early diagnosis and treatment (4-weekly Benzathine penicillin). The multidisciplinary health-care workers, including medical officers, pharmacists, laboratory technicians, health supervisors and multipurpose health workers, are oriented to RF/RHD surveillance and case registration. Patients reporting to either a general outpatient department or referred by health workers or schoolteachers are examined by doctors who follow standard diagnostic procedures in accordance with the Revised Jones criteria 2015 for confirmation of diagnosis and registration of the case [39]. Clinical history and examination by doctors are used to diagnose cases with RHD. Suspected cases are confirmed by the cardiologist in a tertiary care hospital using echocardiography. The confirmed cases are administered benzathine pencillin in the registry setting and are followed up every four weeks.

However, such registries are currently not functional across many regions of the country. A major barrier to RF/RHD registry-based programs in India remains the lack of a continuous supply of benzathine penicillin. Therefore, adequate supply of Benzathine penicillin is urgently needed as an area of public health system strengthening. A recent meta-analysis by Abrams et al 2020 also showed that secondary prevention led to decrease in incidence of RF recurrence (6.4 to 0.4 per 1000), hospitalization rate (41.1% to 8.3%) and prevalence of RF/RHD (8.0 to 2.0 cases per 1000) along with 86.1% decline in cost of managing the disease [51]. Hence, secondary prophylaxis should be promoted as a means of achieving better quality of life among RF/RHD patients and reducing the burden of this preventable cardiac disease.

Additionally, the Central government has introduced *Ayushman Bharat Pradhan Mantri -Jan Arogya Yojana* (ABPM-JAY) which is a large tax-funded national health insurance scheme for the provision of secondary and tertiary care services in public and private hospitals [52]. The scheme covers the component of tertiary prevention of RF/RHD by providing reimbursement to RHD patients undergoing surgical procedures like valve replacement surgery and balloon valvotomy.

The HRQOL of RF and RHD patients was found to be less in households with higher annual household income. This could be because people with higher incomes tend to have better access to health care, higher utilisation of health services and lower thresholds of discomfort than those with lower incomes and who live in poverty. As HRQOL is a subjective health outcome, people with different education levels can perceive its dimensions differently, particularly with regard to psychological functions. As with other studies, the present study found better HRQOL among patients who have attained some education as compared to those with lower literacy [53, 54].

A pertinent strength of the present analysis is that all participants were RF and RHD patients themselves, in contrast to some earlier studies where parents were asked to report health states of behalf of their children [42, 43]. As a result, the utility scores obtained via interviewing parents of affected children might not depict the true picture. The present study depicts comparatively more accurate representation of HRQOL of RF and RHD patients owing to the large sample size of the study covering a range of disease severity and treatment groups.

We would like to acknowledge that there are certain study limitations. Firstly, the study findings might not be generalizable to communities beyond these regions as the valuation of HRQOL is strongly influences by culture, ethnicity, age and region. However, our study sample was drawn from a northern population based RF-RHD registry and the population characteristics are similar to the overall characteristics of RF/RHD patients residing in other parts of the nation. Secondly, utility scores were calculated using value set from another country which might not represent actual perception of Indian population. However, it is worthwhile to mention that value set for Indian population has not been prepared so far, necessitating the use of value set from another country [35–37]. As per standard recommendations in selecting other country value set to be used for converting local health states to utility scores, Thailand appears to be the most appropriate among the countries with a value set [55, 56]. Moreover, the draft Indian reference case for undertaking HTA in India recommends using the Thailand value-set to calculate quality of life index scores [38]. Another limitation is the fact that the analysis by severity-treatment category involved nine strata, such that the precision within small groups was not optimal and apparent differences did not always reach statistical significance.

## Conclusion

It is worthwhile to mention that in contemporary clinical practise, which is predominantly value-based, the true measures of quality of care are the outcomes that matter to patients. When these outcomes are measured and reported, it fosters improvement and adoption of best practices, thus upstreaming the standards of clinical care. Efforts to improve HRQOL of RF and RHD patients should focus primarily upon ameliorating pain and implementation of secondary prevention strategies for reducing the progression from ARF to RHD and prevention of RHD-related complications. As the present study generated utility scores for RF and RHD patients in the local population, its results may be used for conducting cost-effectiveness analysis of the interventions for the prevention and control of RF and RHD in India. However, further studies are needed to develop a local EQ-5D tariff value-set in order to facilitate evidence-informed decision making in India.

## Supporting information

S1 Datasheet (XLSX)

S2 Datasheet (XLSX)
